# Analysis of influencing factors and prediction of falls among rural older adults in China based on a nomogram model

**DOI:** 10.3389/fpubh.2026.1781812

**Published:** 2026-03-10

**Authors:** Yan Wu, Shangci Cao, Pengcheng Wan, Yuran Liu, Yaodong Zhao, Yujie Chen, Yi Li, Hong Ding

**Affiliations:** 1 Department of Health Service Management, School of Health Management, Anhui Medical University, Hefei, Anhui, China; 2 Anhui Provincial Center for Disease Control and Prevention, Hefei, Anhui, China

**Keywords:** falls, influencing factors, nomogram, older adults, prediction model, rural areas

## Abstract

**Objective:**

To explore rural fall determinants and build a nomogram for high-risk identification. This study aims to preliminarily identify high-risk individuals and provide reference for subsequent intervention research.

**Methods:**

Stratified multistage cluster sampling was used: one city from each of the northern, central, and southern regions of Anhui Province, one county per city, and six villages per county (18 villages total). Township hospital staff and village committee staff helped recruit participants who completed a face-to-face questionnaire and physical tests. 1,546 rural adults aged ≥60 years were enrolled. Inclusion: ≥60 years, local residence ≥6 months, communicable. Exclusion: severe illness or bedridden. A fall was explicitly defined as “an event in which the participant unintentionally came to rest on the ground, floor, or a lower level,” excluding incidents caused by sudden paralysis, stroke, or violent impact. Participants were randomly split 8:2 into training (*n* = 1,208) and validation (*n* = 338) sets. Univariate tests (Mann–Whitney/Kruskal–Wallis) screened variables; those with *p* < 0.05 entered multivariate logistic regression with backward stepwise selection to build the nomogram.

**Results:**

The observed fall prevalence was 24.34% in the study population. From the univariate and multivariate analyses of the training set, five variables were identified: age, anxiety, frailty, living arrangement, and frequency of coarse grain consumption. These variables were incorporated into the nomogram model, which exhibited an area under the ROC curve (AUC) of 0.7215 (95% CI:0.690–0.753), indicating good discriminative performance. The calibration curve demonstrated high calibration accuracy. Internal validation of the nomogram model using the validation set yielded an AUC of 0.703 (95% CI:0.644–0.762), reflecting robust discriminative ability. The Hosmer-Lemeshow test indicated good calibration in both the training set (*p* = 0.38) and the validation set (*p* = 0.08) suggesting that there is no statistically significant difference between the predicted and observed probabilities, and confirming good calibration.

**Conclusion:**

The nomogram we built–incorporating age, anxiety, frailty, living arrangement, and frequency of coarse-grain intake–offers a visual risk estimation tool for estimating fall risk in rural older adults. It can readily flag high-risk individuals and provide clues for the follow-up targeted health management or intervention research.

## Introduction

According to data from the National Bureau of Statistics of China, by the end of 2023, the population aged 60 and above in China reached 296.97 million, accounting for 21% of the total population. The population aged 65 and above was 216.76 million, representing 15.4% of the total population, indicating that China has entered a moderately aging society (1). Falls, as a globally recognized major public health issue (2), are common accidental injuries among older adults. Falls have become the leading cause of injury-related deaths among older adults (3, 4), not only significantly impacting the individuals and their families but also imposing a substantial economic burden on society (5). Research shows that approximately one-third of people aged 65 and above and nearly half of those aged 80 and above have experienced falls each year (6–8). The World Health Organization defines a fall as “sudden, not intentional, and unexpected movement from orthostatic position, from seat to position, or from clinical position” (2). Research and guidelines issued by the National Health Commission of China, including the “Fall Prevention Tips for older adults,” indicate that the fall rate, fall-related hospitalization rate, and fall-related mortality rate among older adults in China are gradually increasing.

In China, older adults individuals in rural areas are more likely to experience falls and are at higher risk compared to their urban counterparts (9–11). However, a large number of older adults individuals in rural areas are not aware of their fall risks and the likelihood of falling (12). The occurrence of a fall is not the result of a single factor but rather the interaction of multiple factors. The risk factors for falls can roughly be divided into internal and external factors. Internal factors mainly include age, gender, physical health, mental health, gait, etc. (13–15). External factors mainly include behavior and lifestyle, living environment, and living conditions, etc. (16, 17). The influencing factors of falls may vary across different regions (18–21), potentially affecting the identification of high-risk individuals. Commonly used fall-risk tools—the Berg Balance Scale, Morse Fall Scale, and Tinetti Balance and Gait Scale—focus almost exclusively on motor performance and therefore miss psychological, behavioral, and lifestyle determinants that materially alter risk (22, 23). Rural China–specific fall research has likewise concentrated on descriptive epidemiology and isolated risk factors, without integrating health status, behavior, and lifestyle into a single predictive algorithm (24–27). Older adults in rural regions confront distinct exposures: subsistence farming, uneven terrain, scarce health-care resources, and social–dietary patterns that may modify fall risk in ways not captured by urban-derived models. Nomograms translate multiple predictors into an intuitive, individualized probability and are increasingly used for clinical risk stratification. In fall prevention, Stalenhoef et al. (28) built a recurrent-fall model in 2002, and Jia et al. (29) constructed a fall prediction model based on urban samples, but its applicability in rural scenarios may be insufficient. Therefore, this study aimed to develop a practical and easy-to-use nomogram that integrates health, behavioral, and lifestyle factors specifically relevant to rural Chinese older adults, facilitating individualized fall risk estimation in resource-limited settings.

## Methods

### Study design and participants

From July 2023 to March 2024, we carried out a cross-sectional survey across Anhui Province using stratified multistage cluster sampling. One city was randomly drawn from the northern, central, and southern physiographic zones of the province to guarantee geographic representation. Within each selected city, one county was then randomly chosen, and six villages were randomly selected from each county’s administrative list, yielding 18 villages in total. All permanent residents aged ≥ 60 years in these villages were screened for eligibility. Inclusion criteria: (1) age ≥ 60 years; (2) registered residence in the sampled village for ≥ 6 months; and (3) adequate communication. The exclusion criteria included: (1) severe physical or mental illness that precluded participation in the survey; (2) bedridden status. Trained interviewers from the research team, accompanied by township hospital staff and village committee staff, went to the homes of potential participants to contact them. During the visit, interviewers explained the study in detail, confirmed the eligibility criteria, and obtained written informed consent before starting data collection. This study was approved by the Institutional Review Board/Ethics Committee of Anhui Medical University (Approval Number: 83244655). All procedures performed in studies involving human participants were in accordance with the ethical standards of the institutional and/or national research committee and with the 1964 Helsinki declaration and its later amendments or comparable ethical standards. Written informed consent was obtained from all individual participants included in the study.

### Sample representativeness assessment

To assess the representativeness of our sample for the target population (rural older adults in Anhui Province), we compared its key sociodemographic characteristics with national benchmark data. Specifically, we referenced the indicators for rural older adults published in the Fifth Sample Survey on the Living Conditions of China’s Urban and Rural Older Adults (2021) (30). In our sample, the sex distribution (45.5% male, 54.5% female) was similar to the national rural profile (49.1% male, 50.9% female). Although the proportion of adults aged 70 and above in our sample (65.7%) is higher than the national rural older adults proportion (45.9%). This difference is in line with the backdrop of population aging in Anhui Province. According to the latest population census data, the population aged 60 and above in Anhui Province accounts for 18.79% of the total population (31). The illiteracy rate in our sample (57.1%) was lower than the national proportion of rural older adults with an education level of elementary school or below (74.9%). The proportion of older adults living alone in our sample (20.0%) was also close to the national Figure (16.3%). Overall, these comparisons indicate that our sample captures the key demographic features of the rural older adult population in a rapidly aging region. Combined with the stratified multistage cluster random sampling method, the sample is considered representative of the sampled rural communities in northern, central, and southern Anhui Province.

### Data collection and measures

Following a comprehensive review of relevant literature and consideration of the unique characteristics of rural older adults in China, a structured questionnaire was developed. This questionnaire encompassed socio-demographic characteristics, physical and mental health status, behavioral and lifestyle factors, as well as the occurrence of falls.

#### Socio-demographic and health characteristics

Specifically, socio-demographic data were collected as follows: gender (male/female); age, categorized into three groups: 60–69 years, 70–79 years, and ≥80 years; educational level (illiterate, primary school, junior high school and above); marital status (married, widowed, divorced, and single); living arrangement (living alone, living with spouse, living with children, and living with others); and employment status (working full-time, working part-time, engaged in housework, not working).

#### Physical and mental health status

Objective measurements included height, weight, and blood pressure. Grip strength was measured using a handheld dynamometer; the maximum value from two attempts with the dominant hand was recorded. Chronic disease status was assessed by self-report of physician-diagnosed conditions, recorded as “yes/no” having a chronic disease. Medication use was measured by asking participants the number of all drug classes they were currently taking; The number of medication types was recorded and classified as 0, 1, 2, or ≥3. Frailty was assessed using the Kihon Checklist (KCL), as detailed below. The primary outcome variable, falls, was collected by self-report in response to the question: “Have you experienced any falls (an unexpected event where you come to rest on the ground, floor, or lower level) in the past 12 months?” The response was recorded as a binary variable (Yes/No). Participants were asked about any falls that had occurred in the previous 12 months and these were recorded, regardless of whether they had occurred where they currently lived or elsewhere.

### Behavioral and lifestyle factors

These included taste preference (light; moderate; salty), smoking status (current smoker; former smoker; never smoked), alcohol consumption (current drinker; former drinker; never drank), and frequency of whole grain consumption (<1 day/week; 1–2 days/week; 3–5 days/week; 6–7 days/week). Labor intensity was categorized into light, moderate, and heavy based on participants’ self-reported primary daily activities. Visual blurriness was assessed via a binary question: “Do you frequently experience blurred vision?” (Yes/No).

### Measurement of anxiety

The Generalized Anxiety Disorder Scale (GAD-7) was utilized to assess anxiety levels. This 7-item instrument employs a 4-point Likert scale, with response options ranging from 0 (“not at all”) to 3 (“nearly every day”). Total scores range from 0 to 21, and established cut-off values categorize anxiety severity as none (0–4), mild (5–9), moderate (10–14), or severe (15–21). The GAD-7 has been shown to exhibit good reliability and validity in prior studies (32, 33).

### Measurement of depression

The Patient Health Questionnaire-9 (PHQ-9) was utilized to assess depression severity. This 9-item instrument employs a 4-point Likert scale, with response options ranging from 0 (“not at all”) to 3 (“nearly every day”), and total scores ranging from 0 to 27. Scores are interpreted as follows: no depression (0–4), mild depression (5–9), moderate depression (10–14), moderately severe depression (15–19), and severe depression (20–27). The PHQ-9 is a reliable and valid tool for older adult populations (34). In the present study, the Cronbach’s *α* coefficient for the PHQ-9 was 0.89 (35), indicating excellent internal consistency.

### Measurement of frailty

Frailty was assessed using the Kihon Checklist (KCL), which has been translated into Chinese by Scholar Wang (36, 37). This 25-item instrument comprises seven dimensions, with responses coded as “Yes” or “No.” Total scores range from 0 to 25, with a cut-off score of ≥8 indicating frailty. The KCL has demonstrated good reliability and validity, with reported Cronbach’s *α* coefficients ranging from 0.787 to 0.876 across diverse older adult populations (38, 39). Specifically, among Chinese older adults, the overall Cronbach’s α coefficient has been reported as 0.882 (37).

### Measurement of sleep quality

Sleep quality was assessed using the Pittsburgh Sleep Quality Index (PSQI) (40). This instrument evaluates subjective sleep quality over the preceding month and comprises seven components: subjective sleep quality, sleep latency, sleep duration, habitual sleep efficiency, sleep disturbances, use of sleeping medication, and daytime dysfunction. Each component is scored on a 0–3 scale, with the global PSQI score ranging from 0 to 21. A total score >7 is indicative of poor sleep quality or sleep disturbance (41). The PSQI has been validated and demonstrates good reliability and validity among rural older adult populations (42).

### Data management and statistical analysis

Data were entered twice using EpiData 3.1 to minimize entry errors. A questionnaire was considered invalid when more than 10% of the data were missing. When the data missing in the questionnaire was less than 10%, the mean or median of the index was used to fill in according to the variable type and distribution characteristics of the missing data. Statistical analyses were performed using SPSS 26.0 and Stata 16.0. To facilitate the development and validation of a predictive model for falls, the total study dataset (*N* = 1,546) was randomly divided into a training set (*n* = 1,208, 80%) for model development and a validation set (*n* = 338, 20%) for internal validation (43, 44). Frequencies and percentages were used to describe categorical variables. In the training set, univariate analyses were conducted to identify factors associated with falls using the Mann–Whitney *U* test (for two-group comparisons) and the Kruskal–Wallis *H* test (for multi-group comparisons). After univariate analysis, to eliminate the influence of multicollinearity among independent variables on the stability of the model, we calculated the variance inflation factor (VIF). It is generally believed that a VIF < 10 indicates that multicollinearity issues do not affect subsequent modeling. All variables with a *p*-value < 0.05 in the univariate analyses were subsequently entered into a multivariate binary logistic regression model to identify independent risk factors for falls. The results were presented as odds ratios (ORs) with 95% confidence intervals (CIs). The independent influencing factors identified in the final multivariate model were used to construct a nomogram prediction model using Stata 16.0. The model’s discriminative ability was evaluated by calculating the area under the receiver operating characteristic curve (AUC) in both the training and validation sets. The model’s calibration, or the agreement between predicted probabilities and observed outcomes, was assessed using the Hosmer-Lemeshow goodness-of-fit test and visually represented by calibration curves. A two-tailed *p*-value of < 0.05 was considered statistically significant for all analyses.

## Results

### Basic characteristics and fall prevalence of participants

Table 1 details the prevalence of falls among older adults in rural China. Among the 1,208 participants in the training set, 294 reported experiencing at least one fall in the past year, resulting in a fall prevalence of 24.34%. The prevalence of falls varied across age groups: 22.22% in the 60–69 age group, 28.94% in the 70–79 age group, and 15.71% in those aged 80 and above. The prevalence was 20.55% among males and 25.71% among females.

**Table 1:** Prevalence of falls in rural older adults.

Variable	Fall		Total
	Yes	No	
Total	294(24.34%)	914(75.66%)	1,208
Age (years)			
60–69	92(22.22%)	322(77.78%)	414
70–79	169(28.94%)	415(71.06%)	584
≥80	33(15.71%)	177(84.29%)	210
Gender			
Male	113(20.55%)	437(79.45%)	550
Female	181(27.51%)	477(72.49%)	658

Table 2 presents the general demographic characteristics of the respondents and univariate comparisons between fallers and non-fallers for all collected variables. There were 550 males (45.57%) and 658 females (54.43%). Age distribution was as follows: 414 individuals (34.27%) were aged 60–69, 584 individuals (48.34%) were aged 70–79, and 210 individuals (17.39%) were aged 80 and above. More than half of the older adults (57.12%) were illiterate. Key factors significantly associated with falls in univariate analysis (*p* < 0.05) included dizziness, gait impairment, anxiety, depression, and frailty, among others. Prior to constructing the final multivariate logistic regression model, we assessed multicollinearity among the candidate predictor variables. Variance inflation factors (VIFs) were calculated for each variable included in the preliminary model. All VIF values were well below the commonly accepted threshold of 5 (range: 1.05–4.29), indicating that multicollinearity was not a substantial concern in our dataset and that the included predictors were sufficiently independent for regression analysis.

**Table 2:** General characteristics of participants and univariate analysis of factors associated with falls (*N* = 1,208).

Variable	*n*(%)	*Z*/*H*	*P*
Gender		7.93	<0.01
Male	550(45.53)		
Female	658(54.47)		
Age (years)		15.98	<0.01
60–69	414(34.27)		
70–79	584(48.34)		
≥80	210(17.39)		
Education level		5.42	0.07
Illiterate	690(57.12)		
Primary	355(29.39)		
Junior and above	163(13.49)		
Married status		5.28	0.52
Married	896(74.17)		
Divorced	4(0.33)		
Widowed	271(22.43)		
Single	37(3.07)		
Living arrangement		10.25	0.02
Living alone	242(20.03)		
Living with spouse	678(56.13)		
Living with children	98(8.11)		
Living with others	190(15.73)		
Working status		4.74	0.19
Normal work	294(24.34)		
Half work	223(18.46)		
Housework	327(27.07)		
Do not work	364(30.13)		
Labor intensity		13.36	<0.01
Light	813(67.30)		
Normal	289(23.92)		
Heavy	106(8.78)		
BMI		0.15	0.93
Low body weight	92(7.62)		
Normal weight	593(49.09)		
Overweight	523(43.29)		
Grip strength		6.12	0.01
Normal	228(18.87)		
On the low side	980(81.13)		
Anxiety		45.05	<0.01
None	833(68.96)		
Mild	212(17.55)		
Moderate	80(6.62)		
Severe	83(6.87)		
Depression		43.56	<0.01
None	786(65.06)		
Mild	206(17.05)		
Moderate	96(7.95)		
Severe	64(5.30)		
Very severe	56(4.64)		
Dizzy		22.63	<0.01
<1time/week	513(42.50)		
1–2times/week	313(25.93)		
3–4times/week	142(11.77)		
≥5times/week	240(19.80)		
Eye blur		8.71	0.01
No	203(16.82)		
Light	638(52.86)		
Severe	367(30.32)		
Chronic diseases		4.46	0.04
Yes	888(73.49)		
No	320(26.51)		
The number of medication types		27.08	<0.01
0	474(39.27)		
1	267(22.12)		
2	189(15.66)		
≥3	278(22.95)		
Frailty		71.05	<0.01
No	512(42.42)		
Yes	696(57.58)		
Gait		4.75	0.03
Normal/Bed rest	997(82.60)		
Weak	176(14.50)		
Impaired	35(2.90)		
Sleep quality		7.67	0.01
Bad	573(47.35)		
Good	636(52.65)		
Taste preference		4.41	0.11
Light	368(30.49)		
Moderate	617(51.03)		
Salty	223(18.48)		
Family doctor		0.45	0.50
Yes	1,081(89.48)		
No	127(10.52)		
Smoking		5.63	0.06
No	898(74.32)		
Quit smoking	89(7.37)		
Yes	221(18.31)		
Drink alcohol		7.28	0.03
No	867(71.75)		
Quit drinking	76(6.30)		
Yes	265(21.95)		
Frequency of coarse grain consumption		18.83	<0.01
<1 day/week	480(39.77)		
1–2 days/week	377(31.23)		
3–5 days/week	189(15.58)		
6–7 days/week	162(13.42)		

BMI, Body Mass Index.

### Multivariate analysis of factors influencing falls in the training set

Table 3 presents the results of the binary logistic regression analysis, which includes only variables retained in the final multivariate model after variable selection. The results showed that age, anxiety, frailty, living arrangement and frequency of coarse grain consumption were the independent influencing factors of fall (*p* < 0.05).

**Table 3:** Multivariate analysis of factors associated with falls among rural older adults.

Variable	*β*	SE	Wald *χ*^2^	*P*	OR(95%CI)
Age (years)					
≥80 (Ref)					
70–79	0.96	0.22	18.50	<0.01	2.62(1.69 ~ 4.06)
60–69	0.90	0.24	13.48	<0.01	2.45(1.52 ~ 3.96)
Anxiety					
None (Ref)					
Mild	0.37	0.19	4.08	0.04	1.45(1.01 ~ 2.09)
Moderate	0.36	0.27	1.86	0.17	1.44(0.85 ~ 2.42)
Severe	0.86	0.25	11.42	<0.01	2.36(1.44 ~ 3.89)
Frailty					
No (Ref)					
Yes	1.27	0.18	49.82	<0.01	3.55(2.50 ~ 5.05)
Living arrangement					
Living alone (Ref)					
Living with spouse	0.42	0.20	4.24	0.04	1.52(1.02 ~ 2.27)
Living with children	0.90	0.29	9.44	<0.01	2.45(1.38 ~ 4.35)
Living with others	0.66	0.25	6.70	0.01	1.93(1.17 ~ 3.17)
Frequency of coarse grain consumption					
<1 day/week(Ref)					
1–2 days/week	−0.67	0.26	6.51	0.01	0.51(0.31 ~ 0.86)
3–5 days/week	−0.62	0.23	7.71	0.01	0.54(0.35 ~ 0.83)
>5 days/week	−0.47	0.21	5.06	0.02	0.63(0.42 ~ 0.94)

(Ref) indicates the reference category.

### Construction of the nomogram

**Figure 1:**
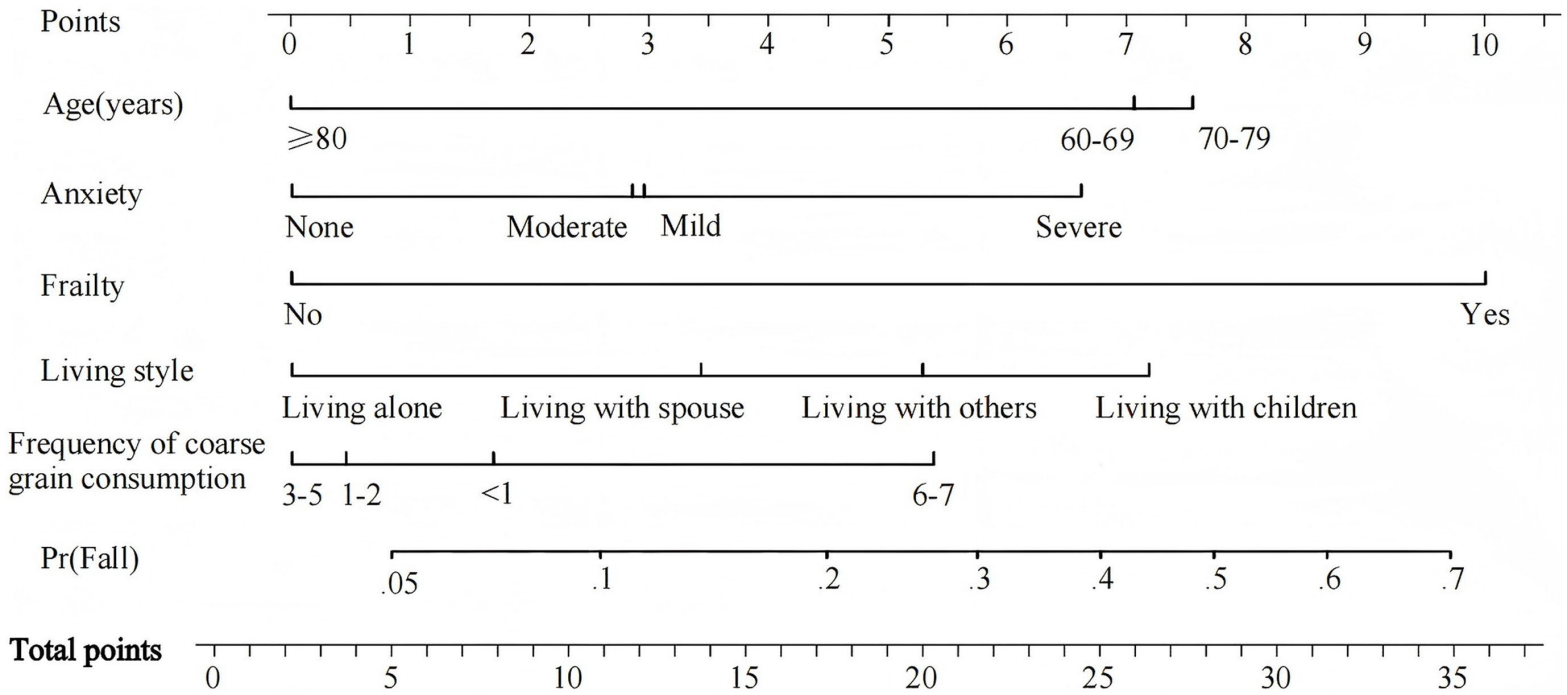
Nomogram for the prediction of fall risk.

To visually represent the predictive effectiveness of the model, a nomogram model for falls among the older adults in rural China was constructed based on the variables identified through multivariate regression analysis (Figure 1). The application of the nomogram is as follows: the total score for fall risk factors ranges from 0 to 35 points, corresponding to a fall rate range of 0.05 to 0.7. By summing the scores vertically corresponding to the first row for each of the five indicators, the total score can be obtained, providing an intuitive estimate of the probability of falls among the older adults in rural areas of China. The higher the total score, the higher the fall rate.

**Figure 2:**
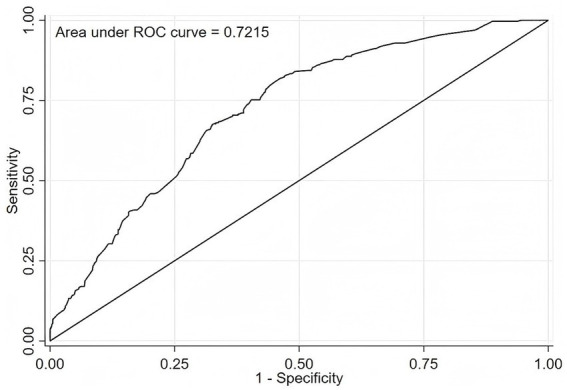
ROC curve of the nomogram in the training set.

Figure 2 shows the ROC curve of the nomogram model in the training set, which has AUC = 0.7215 (95% CI: 0.690–0.753), indicating that the model has a good differentiation; when the model is used in the validation set, the AUC = 0.703 (95% CI:0.644–0.762), indicating that the column nomogram mode also has a good differentiation in the validation set.

**Figure 3:**
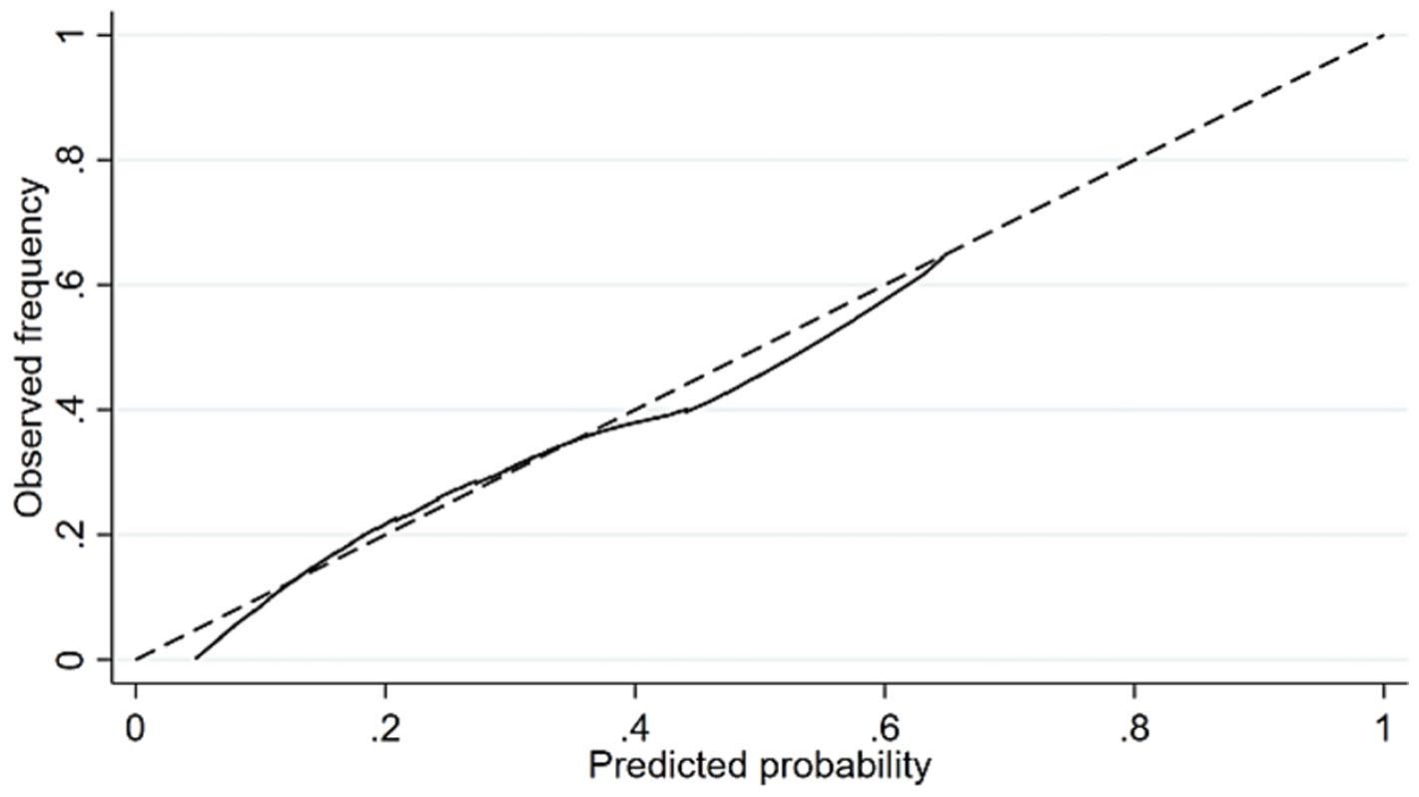
The calibration curve of the nomogram.

Figure 3 presents the calibration curve of the nomogram. As shown in the figure, the predicted probability of falls by the nomogram matches the actual probability quite well. The Hosmer–Lemeshow test result for the training set is *p* = 0.38, indicating a good fit for the training set. The Hosmer–Lemeshow test result for the validation set is *p* = 0.08, suggesting a good fit for the validation set.

## Discussion

The survey results indicate that the fall rate among older adults in rural China is 24.34%. This figure, while higher than previously reported rates for rural older adults in China, aligns with the current trend of increasing fall rates among the older adults (45–47). The multivariate analysis identified age, anxiety, frailty, living arrangement, and frequency of coarse grain consumption as significant factors influencing falls among the older adults in rural China. Based on these factors, we developed a nomogram model to aid in the screening of high-risk fall populations and provide a scientific basis for fall prevention interventions.

The lower risk score assigned to the ≥80 age group in the nomogram appears inconsistent with some literature highlighting increased risk with age (48, 49). This finding requires cautious interpretation and may be explained by several factors. First, survivorship bias likely plays a role: the community-dwelling oldest-old in our sample represent a select, healthier cohort, as their frailer, higher-risk counterparts may have been institutionalized or deceased. Second, reduced exposure to high-risk activities due to age-related declines in mobility and physical capacity lowers the frequency of falls despite potentially high underlying vulnerability. Finally, the smaller sample size in this stratum (*n* = 210) limits the precision of our estimate relative to younger, larger groups. Therefore, the nomogram’s low score for this group should not be interpreted as low intrinsic risk, but rather as reflecting a combination of selective survival, constrained activity, and statistical uncertainty. This study also identified a dose–response relationship between anxiety severity and fall risk: compared to older adults without anxiety, those with mild anxiety had a 1.45-fold increased risk of falls (OR = 1.45, 95% CI: 1.01–2.09), while this risk escalated to 2.36-fold (OR = 2.36, 95% CI: 1.44–3.89) among those with severe anxiety. This finding is consistent with prior research (50). The observed dose–response relationship suggests a strong link between anxiety and fall risk, which may be explained through several potential pathways. From a biological standpoint, it is plausible that anxiety can induce hypervigilance and exaggerated fear responses, potentially disrupting normal gait and postural control. Neurobiologically, anxiety is linked to hypothalamic–pituitary–adrenal (HPA) axis dysregulation and elevated cortisol levels—changes that could negatively impact muscle metabolism and cognitive functions, such as attention and executive control (51), thereby potentially contributing to an elevated risk of falls. This aligns with the “fear avoidance model” (52), which posits that anxiety about falling may paradoxically heighten risk by altering movement patterns. However, these mechanisms are speculative in the context of our data and require verification in studies incorporating biomarker assessment. Rural-dwelling older adults are more susceptible to anxiety than their urban counterparts (74); this not only impairs their mental health (e.g., difficulty concentrating, nervousness, insomnia) but also further compromises physical health and elevates fall risk (24, 53).

The findings of this study indicate that frailty is an independent risk factor for falls among rural-dwelling older adults, with an odds ratio (OR = 3.55, 95% CI: 2.50–5.05) higher than that of other variables (e.g., age and anxiety). This highlights frailty’s core role in fall occurrence, consistent with prior research (54, 55). However, frailty is not a unidimensional physiological decline but a comprehensive manifestation of multisystem functional deterioration—one that significantly elevates fall risk through multiple mechanisms. The strong association between frailty and falls (OR = 3.55) may be mediated through multiple interconnected pathways. Frailty typically encompasses physiological impairments such as reduced balance, gait abnormalities, and decreased muscle strength. It is conceivable that these impairments could directly compromise postural control and the ability to respond to unexpected events, thereby elevating fall risk. In addition, frail individuals are more prone to subjective symptoms such as dizziness and unsteady walking, which may result from vestibular system degeneration, blood pressure regulation disorders or degenerative changes in the nervous system, further interfering with the stability and safety of their daily activities (56, 57). Fear of falling, as a common psychological response among frail older adults, may initiate a vicious cycle of reduced activity and further functional decline (58, 59). Although not directly measured in our study, the sections of the Kihon checklist used to measure frailty that are related to gait and balance may reflect certain aspects of this degenerative process. In addition, most older adults in rural areas are engaged in agricultural labor or household activities. Their living environment is complex, the terrain is uneven, and the labor intensity is high. In the absence of auxiliary facilities or protective measures, the weak are more likely to fall during work or daily activities (60, 61). Therefore, frailty is not only a manifestation of physiological vulnerability but also closely related to environmental exposure behavior. While labor intensity was associated with falls in univariate analysis, it did not remain an independent predictor in the multivariable model. This suggests that its effect may be confounded by or operate through the more fundamental factors of age and frailty, which were retained as the key predictors in the final model. It should also be noted that while previous studies links multimorbidity and polypharmacy to increased fall risk (62, 63), these variables were not independent predictors in our final model. This apparent discrepancy can be interpreted in two ways. Methodologically, the high baseline prevalence of chronic conditions (73.5%) in our rural sample may have limited the variability needed to detect a significant independent effect. More importantly, from a conceptual standpoint, the effect of multimorbidity and polypharmacy on fall risk is likely mediated through the broader syndrome of frailty, which was the strongest predictor in our model (OR = 3.55). Frailty, as measured by the Kihon Checklist, encapsulates the cumulative physiological decline, functional limitation, and vulnerability that result from multiple chronic conditions. Therefore, in our multivariate analysis, frailty may have statistically “captured” the risk pathway associated with comorbidities, rendering the simple count of diseases or medications non-significant. It is also important to note that although factors associated with falls (e.g., dizziness and gait) in the univariate analysis were not included in the final multivariate model, this may be due to the mediating effect of frailty status. Future research should be able to explore these potential pathways through formal mediation analysis.

Contrary to the assumption that living alone increases risk, our study found that those living with others had a higher risk. This finding aligns with previous research (64), although some studies suggest that older adults living alone may face a lack of care, nutritional deficiencies, and challenges in recognizing physical aging, which could also increase fall risk (65). In this study, rural older adults generally have harmonious neighbor relationships, which may mitigate differences in mental health between those living alone and those living with others (66). This could be because individuals requiring co-residence may already have underlying health impairments or functional limitations that necessitate assistance with daily activities. In close-knit rural communities, older adults living alone might be those who are physically more robust and capable of independent living, and they may also benefit from informal community support systems that mitigate isolation (67, 68). The study also found a relationship between fall risk and the consumption of coarse grains. Moderate consumption of coarse grains can promote detoxification, provide essential vitamins and minerals, and maintain health (69, 70), potentially reducing fall risk. However, excessive consumption could lead to gastrointestinal issues, reduced appetite, and increased digestive burden, thereby raising fall risk (71, 72).

A nomogram is a simple, visual tool derived from a multifactorial statistical model that offers a concise representation of key influencing factors (73). With China’s population entering a stage of moderate aging—accompanied by growing numbers of older adults and their associated health burdens—there is an urgent need for a comprehensive, user-friendly screening tool to predict fall risk. The nomogram developed in this study integrates multiple factors, including physical activity, mental health, behaviors, and lifestyle. The model’s calibration curve demonstrates strong discriminative ability and calibration performance (23), while internal validation yields promising results—indicating good reproducibility and generalizability of the model.

### Advantages and limitations

While previous studies on falls among rural older adults in China have primarily focused on epidemiological description and isolated risk factors, this study advances the field by developing and validating a practical, nomogram-based prediction tool. A key contribution is the integration of specific behavioral and lifestyle factors relevant to rural living into a single, interpretable model. This tool holds significant practical value for the initial screening of high-risk individuals and provides a reference for designing targeted fall prevention strategies.

Limitations: Firstly, this cross-sectional study design cannot infer causal relationships. Secondly, data on fall incidents were collected via self-report without verification against medical records. This reliance on recall may introduce reporting bias and affect the accuracy of the outcome measure. Thirdly, the internal validation strategy, based on a single random split of the data and data-driven variable selection, may not fully account for model optimism. Although the model demonstrated consistent performance between the training and validation sets in our sample, this approach has inherent limitations in estimating the true generalizability of the predictive model. Consequently, the apparent discriminative ability (AUC) may be optimistic. The clinical applicability and stability of this nomogram must be confirmed through external validation using independent, prospective cohorts from diverse rural settings. Fourthly, as bedridden individuals were excluded, the nomogram is primarily applicable to community-dwelling, ambulatory rural older adults. It cannot be extended to be used for those patients with severe mobility impairments. Fifthly, due to the absence of measurement of variables (such as environmental hazards, specific disease conditions), residual confounding factors may still exist. In the future, it is necessary to conduct prospective studies, adopt objective fall monitoring methods, verify medical records, and conduct more comprehensive and standardized environmental assessments.

## Conclusion

In summary, falls among rural dwelling older adults in China are associated with age, anxiety, frailty, lifestyle, and frequency of whole grain consumption. The nomogram developed in this study holds practical value, indicating that addressing these associated factors may be considered in comprehensive fall prevention strategies, pending prospective validation. This tool can be utilized to screen high-risk populations and guide the implementation of preventive interventions, ultimately improving the quality of life and overall well-being of rural older adults.

## Data Availability

The raw data supporting the conclusions of this article will be made available by the authors, without undue reservation.
